# Efficient self-emulsification via cooling-heating cycles

**DOI:** 10.1038/ncomms15012

**Published:** 2017-04-27

**Authors:** Slavka Tcholakova, Zhulieta Valkova, Diana Cholakova, Zahari Vinarov, Ivan Lesov, Nikolai Denkov, Stoyan K. Smoukov

**Affiliations:** 1 Department of Chemical and Pharmaceutical Engineering, Faculty of Chemistry and Pharmacy, Sofia University, Sofia, Bulgaria; 2 Active and Intelligent Materials Lab, Department of Materials Science & Metallurgy, University of Cambridge, Cambridge CB3 0FS, UK

## Abstract

In self-emulsification higher-energy micrometre and sub-micrometre oil droplets are spontaneously produced from larger ones and only a few such methods are known. They usually involve a one-time reduction in oil solubility in the continuous medium via changing temperature or solvents or a phase inversion in which the preferred curvature of the interfacial surfactant layer changes its sign. Here we harness narrow-range temperature cycling to cause repeated breakup of droplets to higher-energy states. We describe three drop breakup mechanisms that lead the drops to burst spontaneously into thousands of smaller droplets. One of these mechanisms includes the remarkable phenomenon of lipid crystal dewetting from its own melt. The method works with various oil–surfactant combinations and has several important advantages. It enables low surfactant emulsion formulations with temperature-sensitive compounds, is scalable to industrial emulsification and applicable to fabricating particulate drug carriers with desired size and shape.

## Abstract (web summary)

Emulsions are mixtures of liquids which have applications to pharmaceutical, cosmetic and food components. Here Tcholakova *et al*. have developed a low-energy emulsification method which requires a low amount of surfactant, works for temperature-sensitive compounds and has potential for scale-up.

Discovering and understanding new mechanisms of drop fragmentation is an important element in the efforts to develop novel bottom-up approaches for creation of non-equilibrium micro- and nanostructures of increasing complexity^1^,^2^,^3^,^4^,^5^,^6^,^7^. The annual volume of emulsions processed worldwide is in the range of 100 million tons, while classical emulsification procedures are characterized with very low energy efficiency, below 0.01% for micrometre droplets^8^. The efficiencies are even lower for sub-micrometre ones, as high-shear and/or high-pressure mechanical devices are used, generating high temperatures^8^, which contribute to further incompatibility with a number of pharmaceutical, cosmetic and food components. In contrast, self-emulsification methods allow one to create micro- and nanodroplets without any mechanical energy input^9^,^10^,^11^,^12^,^13^,^14^,^15^,^16^,^17^.

In the process of self-emulsification, drops of one liquid are spontaneously formed in another immiscible liquid. Sub-micrometre oil droplets could be nucleated, for example, by reducing the oil solubility in the continuous medium via changing solvents or temperature^15^,^16^,^17^. Reducing the oil solubilization capacity of microemulsions by similar means is another method for generating emulsion droplets^13^,^14^. These methods require oils that are highly soluble in mixtures of water with other polar liquids or in micellar surfactant solutions, which limits their application to polar oils and/or oils with relatively small molecules.

For nonpolar oils with larger molecules, often encountered in practice, the phase inversion self-emulsification techniques were developed^11^,^12^,^13^,^14^. The latter methods rely on change of the preferred interfacial curvature on change of the temperature or surfactant concentration. All surfactant-involving methods, mentioned above, are characterized by the rather high surfactant concentrations needed (ca. 10–20 wt%). New low-surfactant-concentration methods are needed to expand the relevance of these energy-efficient techniques to a number of industrial applications. Recently^18^, oil nanodroplets were generated by bubble bursting at an interface in a new low-energy approach that gives a promise for industrial scale-up^19^.

In the current study we describe an efficient and scalable process of self-emulsification in which we harness small temperature fluctuations to cause repeated spontaneous bursting of the dispersed drops into smaller droplets, in the processes of drop freezing and melting. We have confirmed that the studied phenomenon is rather general, as it has been observed with numerous oil–surfactant combinations, where the oily phase could be alkanes of different chain lengths, alcohols or triglycerides.

## Results

### Main observations

**Figure 1:**
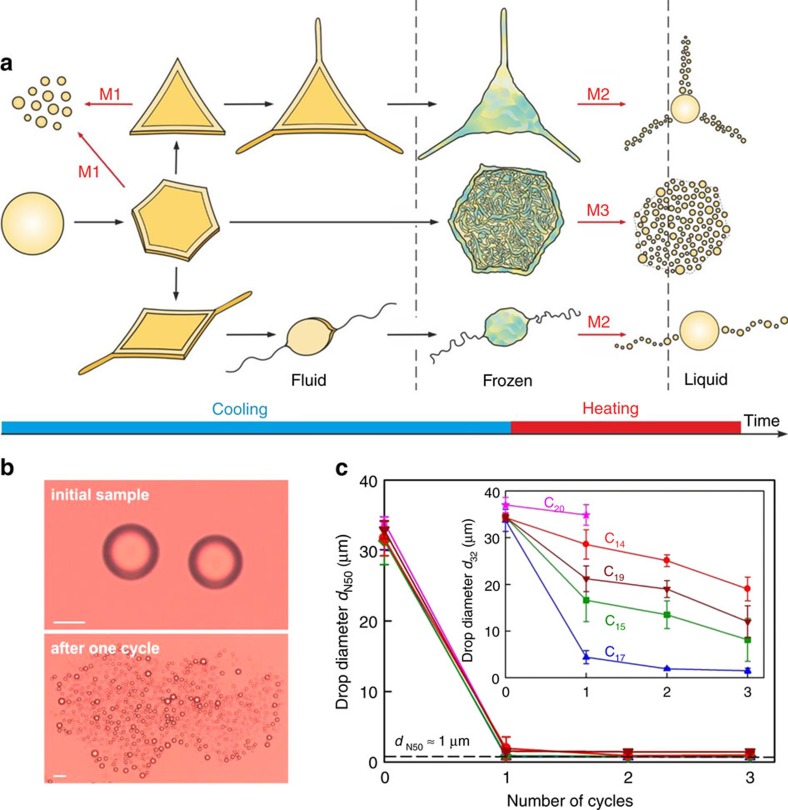
Principle of the self-emulsification method via temperature cycling between freezing and subsequent melting of the droplets in a coarse emulsion leads to spontaneous drop breakup. (**a**) Main drop shape transformations^20^ and related mechanisms of drop fragmentation (M1–3, red arrows) on subsequent cooling and heating of dispersed alkane drops (left to right); Supplementary Fig. 1. In M1 fluid drops spontaneously burst during cooling, while in M2 and M3 frozen particles burst on melting. These mechanisms are illustrated in more details in Figs 2, 3, 4 and explained in the text. (**b**) Microscope images of droplets in a pentadecane emulsion, stabilized by 1.5** **wt% of C_16_SorbEO_20_ surfactant, before and after a cooling/heating cycle (scale bars, 20 μm). (**c**) Typical changes of two measures of the mean drop diameter, *d*_N50_ and *d*_32_, as functions of the number of F/T cycles for alkane-in-water emulsions, stabilized by C_18_EO_20_. Cooling rate 0.2 K min^−1^ and heating rate 1.6 K min^−1^. The error bars are calculated as average values from three independent measurements.

Our process of spontaneous self-emulsification uses relatively low surfactant concentrations (ca. 0.1–3 wt%) and consists of narrow-range temperature cycling around the freezing and melting transitions of the oil drops in an emulsion (Fig. 1 and Supplementary Fig. 1). The dispersed drops are cooled from a liquid state until either droplet breakup or freezing occurs, and subsequently heated back to the initial temperature. These stages are repeated to realize a series of freeze–thaw (F/T) cycles, while the aqueous medium remains liquid. This cycling causes the drops to burst spontaneously into hundreds and thousands of smaller droplets, without any mechanical energy applied to the emulsion (Fig. 1b). We show its applicability to a number of different systems.

The process of self-emulsification was observed by optical microscopy, with emulsions contained in glass capillaries and enclosed in a controlled cooling/heating chamber, see Methods section below^20^,^21^. The drop fragmentation occurs via three mechanisms, none of them observed so far in the self-emulsification context. The first mechanism (M1) occurs during emulsion cooling, when the drops are still fluid, while the other two mechanisms (M2 and M3) occur on melting of the frozen emulsion particles, viz. during heating (Fig. 1a).

We studied a large series of oil-in-water emulsions of linear alkanes, with chain length varying from C_14_ (tetradecane) to C_20_ (eicosane) and melting temperatures varying between *T*_m_=6 °C for C_14_ and 37 °C for C_20_. For emulsion stabilization we used a cationic surfactant cetyltrimethylammonium bromide; anionic surfactant sodium octadecyl sulfate (C_18_SO_4_Na); and two series of nonionic surfactants denoted C_*n*_EO_*m*_ and C_*n*_SorbEO_20_, where *n*=16 or 18 is the number of carbon atoms in the surfactant alkyl chain, *m*=10 or 20 is the number of ethoxy (EO) groups in the hydrophilic head and Sorb denotes a sorbitan ring.

We started all experiments with monodisperse emulsions, prepared by membrane emulsification^22^,^23^,^24^. The drop size distribution was characterized after each F/T cycle by measuring at least 1,000 droplets and calculating the median number diameter, *d*_N50_, and the mean volume–surface (Sauter) diameter, *d*_32_, which represents most closely the biggest drops in the sample^25^. More information is presented in the Methods section below and in the Supplementary Information.

The proposed method is very efficient in generating sub-micrometre droplets, as seen from the examples shown in Fig. 1b,c with C_18_EO_20_ surfactant. Starting with 35 μm drops of different alkanes, the median number diameter, *d*_N50_, becomes smaller than 1 μm after the first F/T cycle for all C_14_ to C_17_ alkanes. At the same time, *d*_32_ decreases rapidly with each F/T cycle for all alkanes with C_15_ to C_19_ chain length (insert of Fig. 1c).

**Figure 2:**
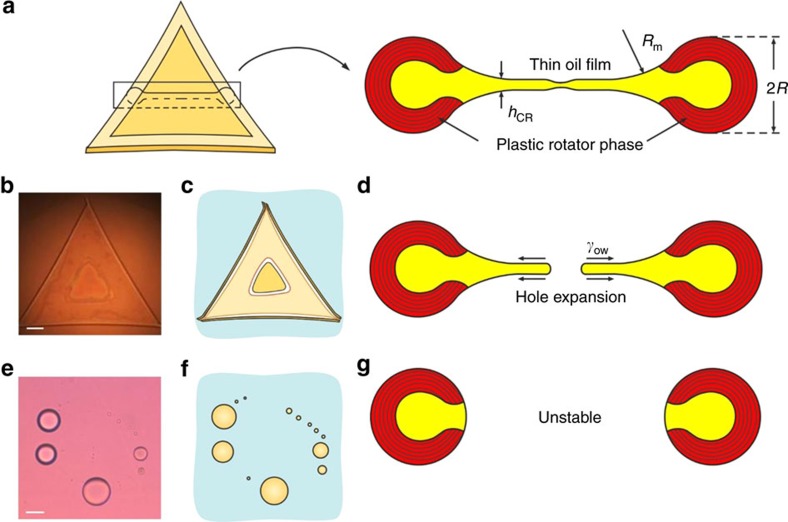
Mechanism 1 involving spontaneous drop bursting on cooling. (**a**) Along their evolution, the alkane platelets may acquire a shape with a thinnest region in their centre (thin water–oil–water film is formed)^20^. (**b**,**c**) Microscope image (**b**) and a schematic presentation (**c**) of a trigonal platelet, which has formed a thin oil film of triangular shape in its centre; (**d**) the rupture of the oil film leads to rapid expansion of the perforating hole, under the action of the oil–water interfacial tension, *γ*_ow_. (**e**–**g**) The toroidal rim, formed after the film breakage, is unstable and breaks into many small droplets, under the action of capillary pressure. The microscope images are with C_16_ drops in C_18_EO_20_ solution, observed in (**b**) reflected or (**e**) transmitted light. Scale bars, 20 μm.

The observed mechanisms of drop bursting are connected to our recent discovery^20^ that cooled alkane drops, dispersed in alkane-in-water emulsions, undergo a series of spectacular shape transformations before their complete freezing (Fig. 1a), if long-chain surfactants are used for emulsion stabilization. These drop shape transformations are driven by the formation of molecular multilayer sheets of plastic rotator phase (on the order of 10^2^ molecules, viz. 100–300 nm thick), composed of self-assembled alkane molecules at the surface of the cooled drops (Fig. 2a)^20^. The transformation of monodisperse drops produced via electrospraying into discotic wax drops was also attributed to the formation of rotator phases in a previous study^26^. Further information about the properties of alkane rotator phases, confined in micrometre droplets and capsules can be found in refs 27, 28, 29, 30, 31, 32, 33, 34, 35, 36, 37. As explained in ref. 20, the multilayers of rotator phase possess a spontaneous curvature and have a bending moment that is sufficiently strong to bend the drop surface against the capillary pressure, which opposes the deformation. The non-spherical drop shapes cause peculiar interfacial instabilities, which we show result in spontaneous drop fragmentation via three distinct mechanisms explained below.

### Mechanism 1 involving platelet puncture and bursting

Upon cooling the spherical drops rapidly flatten to form platelets, due to the formation at the drop periphery of an expanding frame of cylindrical rods, composed of alkane molecules that self-assemble into a plastic rotator phase (Figs 1a and 2a)^20^,^21^. As this frame of finite thickness expands with time and the volume of the droplet is conserved, the frame sucks out molecules from the liquid interior of the platelet, so that eventually the platelet becomes thinnest in its central region, viz. a thin water–oil–water film is formed in the platelet central zone (Fig. 2a–c). This oil film is unstable for some surfactants and punctures in the platelet central region (Fig. 2d). The platelet puncture leads to formation of a toroidal-shaped drop, which is an unstable liquid configuration according to the laws of capillarity. As a result of this instability, the toroidal drop instantly breaks into 3–15 daughter drops (Fig. 2e–g). In some systems stable toroidal drops were obtained due to the high yield strength of the plastic rotator phase^21^, but for most alkane–surfactant pairs the plastic phase was too weak to stabilize these drops after puncture. Typically, 2–6 cycles of consecutive drop flattening, platelet puncturing and fragmentation were observed in a single cooling period, before the drops freeze completely (Supplementary Movie 1). Thus, several hundred daughter droplets are formed from one initial drop in a single cooling period.

The rupture of thin oil films, like those formed in the platelet centre, has never being studied. Therefore, new approaches should be developed to clarify the main factors controlling it. This mechanism was observed only with the systems in which thin flexible layers of rotator phase were formed^21^. The observed film rupture is an important evidence that the thinning platelet central region is not stabilized by a rotator plastic phase, which is present at the platelet periphery, and can be considered as a liquid oil film. This observation allows us to analyse the platelet breakup using the knowledge on liquid film stability, gathered in the context of drop–drop coalescence (viz. for the reverse process), where thin films are formed between two colliding drops or bubbles. Thus, we could ‘borrow' some lead ideas and approaches from previous studies^38^,^39^,^40^,^41^,^42^ to clarify the main factors that control the self-emulsification via Mechanism 1. Unlike aqueous films, which are efficiently stabilized by conventional surfactants or polymers, thin oil films are known to be inherently unstable, due to the lack of electrostatic repulsion and the rather weak steric repulsion between the film surfaces^42^. Under the action of a negative capillary pressure, which sucks the liquid from the film into the surrounding meniscus region, the film thickness *h* would decrease according to the Reynolds equation at a rate proportional to *h*^3^ (refs 38, 39), and would theoretically take a very long time to break. In reality, the liquid films rupture faster and at a larger critical thickness, *h*_CR_, when the van der Waals forces between the two film surfaces become sufficiently strong to overcome the excessive interfacial energy created by a local surface deformation, which leads to film instability and rupture^38^,^39^,^40^. This process is illustrated in Fig. 2a–c and *h*_CR_ is given by equation (1)^40^:


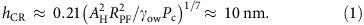


Here *A*_H_≈4 × 10^−21^ J is the Hamaker constant for water–oil–water liquid film, *R*_PF_≈10 μm is the radius of the planar film in the platelet centre, *γ*_ow_≈5 mN m^−1^ is the interfacial tension and *P*_c_≈10^3^ Pa is the capillary pressure that drives the film thinning (estimated as *γ*_ow_/*R*_m_, where *R*_m_ is the radius of curvature of the concave oil–water meniscus around the planar film; Fig. 2a). For a film rupture to occur, it is sufficient that the film thins locally to *h*_CR_, which may be a relatively fast process occurring within seconds and minutes^38^,^39^,^40^.

Thus, the platelets puncturing in their centre indicates that attractive van der Waals forces rupture the oil film and trigger the droplet breakup in this mechanism. This self-emulsification process, occurring via platelet thinning and rupture, can be systematically studied using the variety of methods (both theoretical and experimental), developed in thin film research. Such further studies, for example, following the kinetics of film thinning and shape change, are expected to yield quantitative information about the specific properties of surfactant adsorption layers and the shape fluctuations present in this novel system.

This mechanism is highly efficient to reduce *d*_N50_, as many small drops are generated. It is less efficient in reducing *d*_32_, as the drop fragmentation typically leads to formation of several relatively large ‘daughter' drops, besides the numerous small droplets (Fig. 2e,f). It is more pronounced at low cooling rates (below ca. 1 K min^−1^), as the drops need time to deform and to reach the shape of a concave platelet with a thin central region, before freezing.

### Mechanism 2 involving capillary instability of long fibres

**Figure 3:**
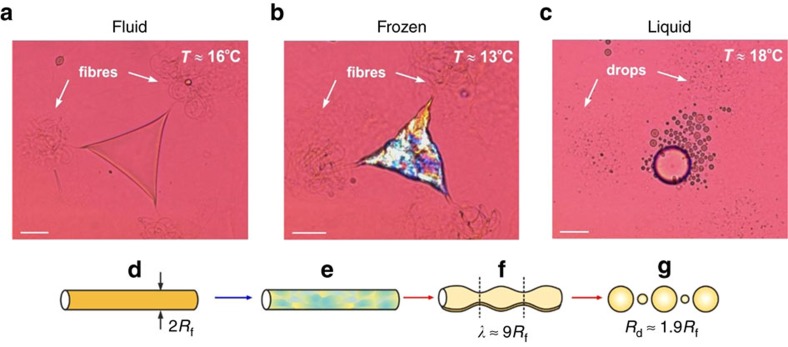
Mechanism 2 involving fragmentation of melting fibres due to a Rayleigh–Plateau instability shown for a trigonal platelet with long coiled protrusions. (**a**–**c**) Illustrative microscope images for C_16_ platelet, stabilized by C_18_EO_20_ (Supplementary Movie 2). (**a**) Fluid triangular platelet freezes (**b**) on cooling and melts (**c**) on heating, with fibre fragmentation; scale bars, 20 μm. (**d**–**g**) Schematic presentation of the main processes leading to fibre fragmentation. (**d**) The fluid and (**e**) the frozen fibres are stable due to the plastic/solid material contained; (**f**) Rayleigh–Plateau instability develops on fibre melting, which leads to (**g**) formation of numerous small droplets from the fibres.

The next stage in the drop shape evolution on cooling is the formation of long fibres (threads) protruding from the platelet corners with acute angles (Figs 1 and 3a)^20^. These fibres are stable on cooling and freezing, due to the elasto-plastic properties of the cylindrical shells of rotator phase, formed at the fibre surface^20^,^21^. The lack of a Rayleigh–Plateau instability^43^,^44^ in the fibres, which would lead to surface undulations and breakup, requires that the capillary pressure created by the fibre interfacial tension, *P*_c_=*γ*_ow_/*R*_f_, has to be counter-balanced by the elasto-plastic properties of the plastic rotator phase. Experimental and theoretical analysis for the stability of gel-like cylinders of soft solid material requires^45^


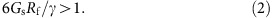


where *G*_s_ is the elastic shear modulus of the gel, *R*_f_≈1 μm is the fibre radius and *γ*≈5 mN m^−1^ is the interfacial tension. Assuming that the fibres in our system are composed of homogeneous material, one estimates the lower limit of the elastic shear modulus of the non-frozen fluid fibres: *G*_s_>*γ*/6*R*_f_≈10^3^ Pa. The latter value corresponds to a soft paraffin wax, close to its melting transition^46^, as one should expect for the system under consideration.

In the moment of fibre melting we observed its fragmentation into numerous small droplets. The breakage mechanism of the melting fibres into equal-size droplets is not obvious, as the frozen fibres contain crystal domains of different sizes (evidenced by their different colours in polarized light) with defects appearing between them, due to the sudden oil shrinkage on solidification. Therefore, one could expect that the different crystal domains could melt into separate oil drops, as described for Mechanism 3 below. However, our measurements with optical microscopy and light scattering showed that in all systems where this was the main fragmentation mechanism, the droplets formed after fibre breakage were with diameter about twice as large as the fibre diameter, without a link to the size of the crystal domains, which is different in the various systems. These results evidence that the melting fibres break in a Rayleigh–Plateau type of hydrodynamic instability, familiar for liquid cylinders, without relation to the specific structural features of the solid fibres, besides their mean diameter^43^,^44^,^45^. This is an indication that the melting oil immediately fills and ‘heals' the defects between the (still) solid domains in the fibres, thus forming a cylindrical liquid jet that fragments into droplets. This ‘healing' does not occur in the systems that break via Mechanism 3.

Depending on the fibres' length, which could be several millimetres if sufficiently slow cooling is used (cooling rate <1 K min^−1^), we observed the formation of hundreds or thousands of micrometre and sub-micrometre droplets from a single mother drop (Fig. 3c and Supplementary Movie 2). Therefore, this mechanism is also very efficient in reducing *d*_N50_. In many of the systems studied, the central body of the platelet melted back into one big drop, which leads to bi-modal emulsions, with two distinct characteristic drop sizes, the small drops being much smaller in diameter and much more numerous (Fig. 3c and Supplementary Fig. 2). The fraction of small droplets increases with each consecutive F/T cycle. Such bimodal distributions are of interest when higher volume fraction packing is required, as the smaller drops or the respective frozen particles could fit into the interstitial space left over when packing together the larger drops/particles.

### Mechanism 3 involving melt-crystal fragmentation

**Figure 4:**
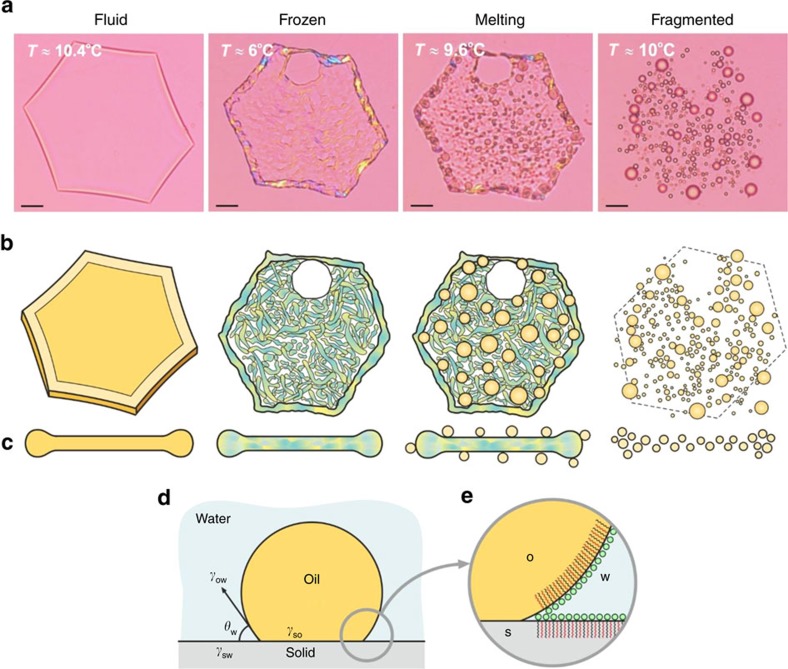
Mechanism 3 involving crystal-melt fragmentation. (**a**) Consecutive images of pentadecane (C_15_) drops in 1.5** **wt% of C_16_SorbEO_20_ surfactant solution, showing from left to right: a fluid hexagonal platelet; a frozen platelet; onset of melting of the frozen platelet; and a particle fragmented into numerous liquid drops after melting (scale bars, 20 μm). (**b**) Schematic presentations in frontal and (**c**) in side view of the images in **a**. (**d**) Dewetting of the solid alkane substrate from its liquid melt (oil) is possible only when the interfacial tension of the solid–water interface is lower than that of the solid–oil interface, *γ*_sw_<*γ*_so_ (equation (3)). (**e**) A dense surfactant adsorption layer, with interdigitated alkane molecules (shown in red), has to form on the solid–water interface to reduce *γ*_sw_ sufficiently for such a dewetting to occur.

Most unexpected was the third mechanism where, for some oil–surfactant combinations, oil droplets can deform and freeze as flat platelets, and on heating, burst (fragment) into numerous small droplets in the moment of their melting. Individual droplets could be traced to the individual alkane crystal domains in the frozen platelets—the latter can be discerned due to their birefringence and various orientations (Fig. 4 and Supplementary Fig. 3). In systems that display this behaviour we noticed that the alkane and surfactant tails were of the same length or the surfactant tail was longer by one or two carbon atoms only. Again, low cooling rates (<2 K min^−1^) are needed to ensure drop deformation and platelet formation during the cooling phase. In contrast, the heating rate had negligible effect—self-emulsification with virtually the same drop size distribution was observed at heating rates up to 5 K min^−1^ (same results were obtained for Mechanism 2). The slower cooling rate, ca. <2 K min^−1^ for hexadecane, required to effectuate the three observed self-emulsification mechanisms, is related to the relatively slow process of rearrangement of the alkane molecules in the plastic rotator phase, as this rearrangement drives the drop deformation needed to trigger the self-emulsification mechanisms.

Microscope observations show that this mechanism is controlled by the dewetting of the crystalline alkane domains (which are still frozen) from the same liquid alkane (just melted) (Supplementary Movie 3). We term this mechanism ‘melt-crystal fragmentation'. The wetting properties are controlled by the energies of the interfaces that meet at the three-phase contact line^47^. In the systems considered the condition for dewetting corresponds to a contact angle *θ*_W_<90°, as measured within the aqueous phase (Fig. 4d). The relation between *θ*_W_ and the interfacial energies of the three interfaces, meeting at the contact zone, is expressed by Young–Laplace equation:


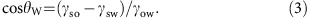


where the subscripts s, o and w denote the solid substrate, liquid oil and water phase, respectively. One sees from equation (3) that dewetting will occur only if *γ*_so_>*γ*_sw_.

The latter condition for dewetting is not easy to achieve when the solid substrate is chemically similar or identical to the wetting liquid oil and the respective interfacial energy is low, *γ*_so_≈3–8 mN m^−1^ (ref. 48). For comparison, *γ*_sw_≈50 mN m^−1^ for the bare interface water-frozen alkane^49^. Therefore, only appropriate surfactants, which form a compact adsorption layer on the solid–water interface and, thus, decrease significantly the value of *γ*_sw_, are able to induce such a dewetting process. As the area per molecule of the surfactants studied is larger than the cross-section of their hydrocarbon tails^50^, we expect that such compact layers could be formed only by including interdigitated alkane molecules^51^,^52^ (Fig. 4e). Only alkane molecules with appropriate chain length could pack well in such mixed adsorption layers and reduce the solid–water interfacial tension to very low values. Note that the respective solid–water interface is created by freezing of a fluid oil drop in the respective surfactant solution, viz. this mixed surfactant–alkane layer is formed before freezing of the solid surface. Thus, we explain the experimental observation that this mechanism is operative only for surfactants with tails that are equal or longer by one or two carbon atoms than the alkane molecules (Supplementary Fig. 4) with the requirement for formation of a closely packed mixed adsorption layer of surfactant+alkane at the solid surface.

Supplementary Movie 3 shows the typical melting of a platelet, where the various crystal domains do not melt simultaneously but within a period of several seconds. This difference in the melting moment for the different domains is probably due to a combination of two related phenomena. First, slight differences in the molecular packing inside the neighbouring domains could lead to differences in the melting temperatures. Second, the process of domain melting consumes enthalpy (due to the high phase transition energy of alkanes), which might reduce locally the temperature in the neighbouring domains. Both mentioned phenomena would lead to differences in the moment of melting for the various crystal domains, as observed experimentally.

This mechanism rapidly reduced the *d*_32_ measure of the drop diameter (the size of the biggest drops), see, for example, the results for C_17_ in Fig. 1c and Supplementary Fig. 4. This is in contrast to other oil–surfactant systems (Fig. 3c and Supplementary Movie 4) in which the alkane melt wets very well the solid crystal domains, so that the platelet thaws into one large drop, possibly surrounded by several very small satellite drops, as one would expect for liquid and solid phases equivalent in chemical composition.

### Relation to other processes

Analysing the melt-crystal fragmentation phenomenon, described above, we realized that it is the basis for an industrial process, used for decades in food technology to separate fats (triglycerides and fatty acids) with higher melting temperature from those with lower melting temperature in natural products such as palm oil, coconut oil and lard^53^,^54^. In this technology, appropriate surfactants are used as wetting agents to hydrophilize the saturated (solid) fats from unsaturated (liquid) oils and transfer the solid crystals into the aqueous phase. The hydrophilization of the solid fat crystals is similar to the one we observe during self-emulsification, though in our case dewetting also occurs for melt and crystals of the same chemical substance.

**Figure 5:**
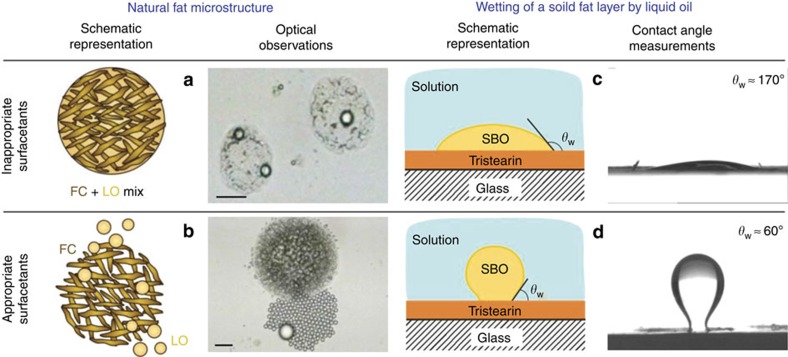
Separation of natural fats into unsaturated (liquid) and saturated (solid) components. (**a**) The typical fat globule consists of a network of saturated fat crystals (FC), impregnated by unsaturated liquid oil (LO). (**b**) With appropriate surfactants one can induce dewetting of the fat crystals by the liquid oil in a type of melt-crystal segregation process (Supplementary Movie 5). (**c**,**d**) This process is controlled by the contact angle of the oily phase on the solid fat substrate. Surfactant mixtures of 0.05** **wt% (in total) LAS+EO7 (linear alkylbenzene sulfonate+polyoxyethylene alkyl ether) at pH=10 are used in these experiments. Upper row is for 3:7 LAS:EO7 ratio, whereas the lower row is for 7:3 LAS:EO7. The fat globules in the microscope images are composed of a 7:3 soybean oil:tristearin mixture (SBO-TS). Scale bars, 20 μm.

We simulated the fat–oil separation process in the lab with a mixture of unsaturated and saturated triglycerides. We observed this process microscopically (Fig. 5a,b and Supplementary Movie 5) and found that the liquid oil fraction, containing unsaturated fats, dewets the solid crystals of the saturated fats and spontaneously disperses in the aqueous phase in a process of self-emulsification, very similar to that reported above for the melting alkane droplets. Note the regular size of the oil droplets, released from the pores formed by the network of fat crystals in this phase separation process (Fig. 5b and Supplementary Movie 5). Measurements of the contact angles of the liquid oil fraction over solid fat substrates confirmed that this process is controlled by the wetting properties of the surfactant+oil pairs (Fig. 5c,d).

From the viewpoint of the colloid and interface science, the process of oil drop release in Fig. 5b resembles closely the process of membrane emulsification^22^,^23^,^24^, both being controlled by the wetting properties of the solid pore walls, with the main difference being that the former occurs spontaneously—no transmembrane pressure is needed to drive it, as it is the case of the membrane emulsification. Thus, we have demonstrated a close mechanistic similarity of the three processes—oil self-emulsification, fat/oil phase separation and membrane emulsification. The underlying wetting phenomena are relevant also to other technologies, in which a phase release from porous materials is included, such as tertiary oil recovery^55^.

### Potential for scale-up

**Figure 6:**
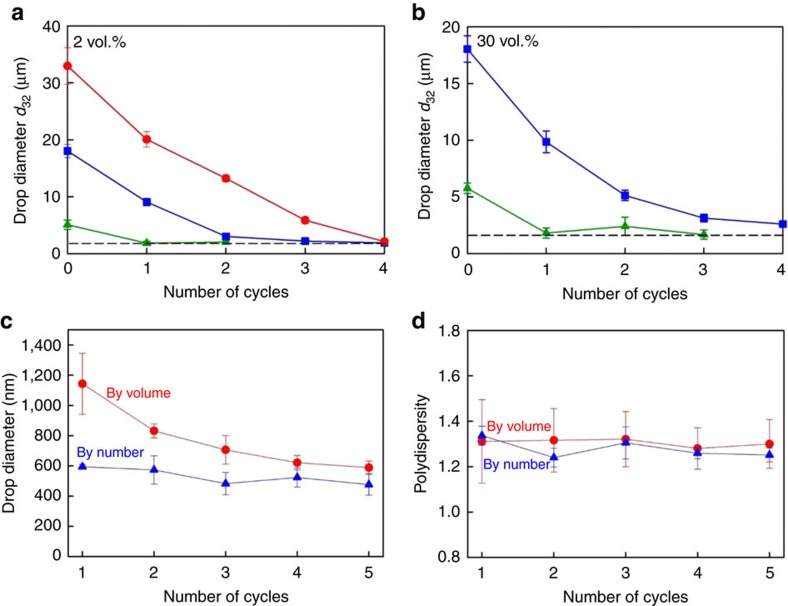
Self-emulsification in bulk emulsions. (**a**,**b**) Volume–surface diameter, *d*_32_, as a function of the number of F/T cycle for C_16_-in-water batch emulsions, stabilized by 1.5** **wt% C_18_EO_20_. Experiments are performed with initial drop diameters of 35, 18 or 6 μm, and at drop volume fractions of 2 and 30 vol.%, as shown in the figures. The horizontal dashed line shows *d*=1.5 μm. (**c**) Mean drop diameters by volume and by number, as measured by DLS, and the respective (**d**) drop polydispersity, for the emulsions with initial *d*_32_≈6 μm. The error bars are calculated from four independent measurements.

To demonstrate that the method for self-emulsification has a potential for scaling-up to industrial emulsification and other technological applications, we performed experiments with batch emulsions. Alternately placing a vial with a coarse emulsion to cool slowly in a refrigerator until a drop freezing, and subsequent tempering back to room temperature, was sufficient to observe an efficient self-emulsification for C_18_EO_20_+C_16_ (Fig. 6). The fragmentation was much faster with smaller initial droplets. The diameter of the droplets formed after each F/T cycle was measured by dynamic light scattering (DLS) (Fig. 6c). Starting with an initial drop diameter of 6 μm, we found that the mean volume diameter after the first cycle was 1.1±0.2 μm and decreased further to 0.6±0.1 μm in the subsequent cycles. The respective mean number diameter was 0.45±0.1 μm. These drop diameters suggest that the smallest drop size is probably determined by the thickness of the plastic rotator phase on the drop surface, ca. somewhere between hundred and several hundred nanometres^20^,^21^. The emulsion polydispersity was characterized by the ratios *σ*_V_=*d*_V84_/*d*_V50_ and *σ*_N_*=d*_N84_/*d*_N50_ (by volume and by number, respectively), which are standard polydispersity measures for log-normal distribution^25^ (see Methods for their definitions). Polydispersity of *σ*_V_≈*σ*_N_≈1.29±0.05 was obtained for all these samples (Fig. 6d). This value corresponds to a relative width of the log-normal distribution curve of ln(*σ*_V,N_)≈0.25 (ref. 25), which is a typical value for emulsions obtained with high-shear or high-pressure homogenizers.

Thus, we have shown that the self-emulsification process, proposed in the current study, is efficient for larger emulsion volumes and fractions. This is important for the development of many technological emulsion systems, including various pharmaceutical, agro, cosmetic and food products^9^,^13^,^14^,^15^. Compounds in such emulsions are often deteriorated by the high mechanical and heat stresses, created in the conventional emulsification devices^15^,^56^. Therefore, new emulsification methods, which are able to disperse temperature-sensitive compounds and are with reduced energy consumption, would advance significantly the current emulsification technologies^10^,^11^,^12^,^13^,^14^,^15^,^16^,^17^,^18^. By flowing the emulsion along repeated pairs of conventional heating and cooling chambers (heat exchangers), the principle of the method (Fig. 1 and Supplementary Fig. 1) allows its straightforward realization in a continuous operational mode, which has a number of advantages and is often the preferred industrial method in comparison with discontinuous batch processing^57^.

## Discussion

The energy efficiency of the emulsification methods is conveniently estimated using the concept of energy density during emulsification, *E*_V_ (refs 8, 56). This is the energy introduced into unit emulsion volume to achieve a desired drop diameter, *d*_32_. The standard high-pressure homogenizers and toothed colloid mills are characterized with *E*_V_≈3 × 10^7^–10^8^ J m^−3^ for drops with *d*_32_ between 3 and 5 μm, and oil volume fraction of 30% (ref. 8). This energy input is needed to create the high inertial and viscous stresses, required to disrupt the oil droplets in the hydrodynamic flow. The vast majority of this energy is dissipated as heat in the active zone of the homogenizer, where the local density of energy dissipation is even two to three orders of magnitude higher than the average values of *E*_V_, quoted above^56^. Simple estimate shows that the total interfacial energy, *E*_S_, of a 30 vol.% oil-in-water emulsion, with 4 μm in diameter droplets and 5 mJ m^−2^ interfacial tension, corresponds to ≈2.3 × 10^3^ J m^−3^. In other words, <0.01% of *E*_V_ only is used to generate the oil–water interface. To obtain smaller droplets is even less efficient, as the drop size in these devices decreases as *E*_V_^−*b*^, with *b* typically taking values between 0.3 and 0.6 (refs 8, 56).

The energy density of our procedure could be estimated from the required temperature variation (±10 °C) and the heat capacity of the emulsion ≈4 × 10^6^ J K^−1^ m^−3^, which gives a first estimate of *E*_V_≈4 × 10^7^ J m^−3^. However, the current heat pumps have a coefficient of performance >4, which means that the same temperature variation could be obtained with about four times less energy consumption by pumping heat out of the cooling chamber, that is, a more realistic estimate is *E*_V_≈10^7^ J m^−3^. The heat removed from the cooling section can be used for heating the subsequent heating section, that is, the same heat pump can be used to control both the cooling and heating compartments in our method. Note that we can obtain sub-micrometre droplets with this energy density (Figs 1c and 6c), viz. our method is comparable in energy efficiency to the optimized, highly efficient high-pressure homogenizers with sharpened edge valve, which produce similar in size drops at similar energy consumption^8^.

Thus, we have discovered an efficient and scalable process of self-emulsification, which is effective at low surfactant concentrations of ca. 0.1–3** **wt% required to ensure a complete coverage of the drop surface with dense adsorption layers. This method does not require any special equipment, besides a simple cooling device. The self-emulsification is based on spontaneous bursting of the dispersed drops into hundreds and thousands of smaller droplets, during freezing and melting, without any mechanical energy applied to the emulsion. In all three mechanisms discovered, the energy of phase transition to the rotator phase and to the solid alkane, accumulated during drop cooling and freezing, is transformed into interfacial energy of the smaller drops in the final emulsion. Sub-micrometre droplets could be generated under appropriate conditions, with a polydispersity that is comparable to that obtained in the industrial high-shear and high-pressure devices^58^, while being significantly larger than that in the microfluidic^59^,^60^,^61^ and membrane emulsification techniques^22^,^23^,^24^.

Using appropriate cooling rates and surfactant–alkane combinations, we observed this phenomenon with all types of surfactants (nonionic, cationic and anionic) and with all alkanes (chain length varied between C_14_ and C_20_) we have tested. Thus, we have confirmed that the studied phenomenon is rather general, as it is not limited to a narrow range of surfactant–alkane pairs. Best results were obtained with the surfactants having a chain length that is slightly longer or similar in length to the alkane molecules (for example, C_18_EO_20_+C_17_, C_16_SorbEO_20_+C_15_, C_16_TAB+C_16_ and C_18_H_37_SO_4_Na+C_16_). For these systems all three mechanisms were operative and the mechanism of melt-crystal fragmentation was particularly efficient for the reasons explained above.

The processes of drop shape transformations and platelet formation, which are essential for the self-emulsification phenomena reported here, were observed with other classes of organic molecules such as triglycerides, alkyl cyclohexanes, linear alcohols and alkenes^21^. Indeed, preliminary experiments demonstrated self-emulsification phenomena by similar mechanisms with alcohols and triglycerides (Supplementary Fig. 5). Thus, we have confirmed that the observed processes of melt-crystal fragmentation and self-emulsification are not limited to linear alkanes and are more general. Further systematic studies are needed to reveal the full range of materials, which could be processed by the proposed method, as well as to optimize the procedure with respect to the minimal drop size and the lowest drop polydispersity, which could be achieved.

The deep understanding of the underlying mechanisms, reported in the current study, opens opportunities to harness these self-emulsification processes for the development of new technological applications. For example, this method is of high potential interest for producing pharmaceutical emulsions and dispersions of temperature-sensitive drugs. Not only the drop size could be reduced at minimal heat and mechanical stresses but also the lipid drug carriers could be frozen in a desired shape^20^,^21^, and surface-modified to achieve selective particle uptake in specific organs^62^,^63^ and/or controlled release from slow dissolution^64^ or from slow enzyme lipolysis in the intestinal fluids and blood plasma^65^.

The irreversibility of the system is interesting also from a thermodynamic point of view and previous efforts have achieved self-emulsification and multiple breakup by generation of reactive chemical species at the oil–water interface^66^,^67^,^68^. By contrast here harness energy from temperature fluctuations in the environment of only a few degrees, and achieve repeated droplet breakup purely from physical transformations. The kinetics and symmetry breaking in the transformations eventually lead to barriers preventing the system from returning to its initial lower-energy state. Controlling and engineering such irreversibility has relevance for understanding of many living and other non-equilibrium systems.

## Methods

### Microscope observations

All optical observations were performed with AxioPlan and AxioImager.M2m microscopes (Zeiss, Germany). For the F/T cycles we used transmitted, cross-polarized white light, with included compensator plate situated after the sample and before the analyser, at 45° with respect to both the analyser and the polarizer. When taking images for determination of the drop size distribution we used transmitted light. The morphological changes in mixed tristearin-soybean oil (TS-SBO) layers, placed in contact with surfactant solutions, were observed also in transmitted white light (Supplementary Fig. 6). Long-focus objectives LD EC Epiplan-Neofluar (Zeiss) of magnification × 20 (numerical aperture (NA) 0.22 and working distance (WD) 12 mm), × 50 (NA 0.55 and WD 9 mm) and × 100 (NA 0.75 and WD 4 mm) were used, as appropriate. Video camera AxioCam MRc (Zeiss) was attached to the microscope with camera adapter 60 N-C 2/3″ × 0.63.

### Procedure for drop F/T cycling for microscope observations

**Figure 7:**
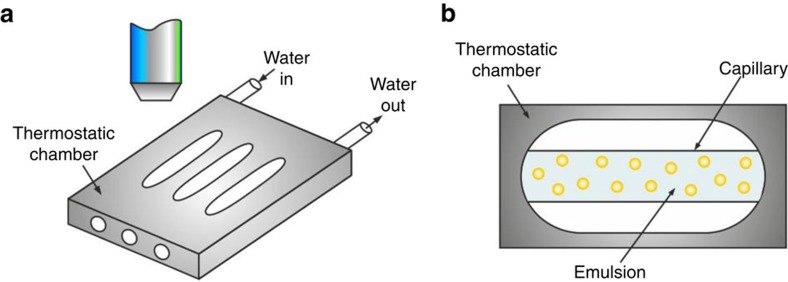
Experimental set-up. (**a**) Schematic presentation of the cooling chamber, made of aluminium, with optical windows cut out, used for microscope observation of the emulsion samples. (**b**) The emulsions studied were contained in glass capillaries, placed in the thermostatic chamber and observed through the optical windows^20^,^21^.

A specimen of the studied emulsion was placed in a capillary with length of 50 mm, width of 1 mm and height of 0.1 mm. The capillary was enclosed within a custom-made metal cooling chamber, with optical windows for microscope observation. The chamber temperature was controlled by cryo-thermostat (JULABO CF30, Cryo-Compact Circulator) and measured using a calibrated thermo-couple probe with an accuracy of ±0.2 °C (Fig. 7).

The accuracy of temperature measurements was ensured by the following procedures: (i) the thermocouple probe was calibrated with a precise mercury thermometer in the respective range of temperatures measured. The deviations were within ±0.1 °C. (ii) The thermo-probe was inserted in one of the orifices of the aluminium thermostating chamber (Fig. 7) and mounted in the position, where a capillary with the emulsion sample would be normally placed for microscope observations. In the neighbouring orifices actual capillaries with emulsion samples were positioned. During an experiment, the thermo-probe temperature was measured and assumed to be the same, as in the neighbouring capillaries with emulsion samples. (iii) After freezing of the emulsion droplets, they were heated back to observe the drop-melting process. The temperature of melting was measured and in all cases it was very close to the melting temperature of the bulk oil, *T*_m_ (±0.2 °C). The latter check allows us to claim that the accuracy of temperature measurements is ±0.2 °C.

F/T cycles of the dispersed drops were performed as follows: (1) the cooling process started with a fixed cooling rate (chosen in the range between 0.1±0.02 and 2±0.1 K min^−1^) from the initial temperature, *T*_0_, which was 15 °C for C_14_ emulsions, 20 °C for C_15_, 22 °C for C_16_, 30 °C for C_17_, 35 °C for C_18_, 40 °C for C_19_ and 45 °C for C_20_. All these *T*_0_ were slightly above the bulk melting temperatures of the alkanes. The temperature in the system was lowered to freeze completely the dispersed oil drops, which corresponded to lowest temperature in the cycle, which was, depending on the system, between 8 and 12 °C below *T*_m_. (2) The drop-melting process was performed at a fixed heating rate (chosen in the interval between 0.2±0.01 and 5±0.2 K min^−1^). The temperature in the system was increased until reaching the initial temperature, *T*_0_, at which all dispersed entities were in the liquid state. Then procedures (1) and (2) were repeated two more times with the same sample to reach three F/T cycles in total. Only the dispersed phase underwent a phase transition during our experiments, while the continuous medium (aqueous surfactant solution) remained liquid.

### Self-emulsification in bulk emulsions

The drop size evolution in bulk emulsions was studied with 15 ml samples placed in glass containers. These samples were first cooled for 2 h in a refrigerator at a temperature of 7 °C to achieve a complete oil drop freezing, followed by melting of the emulsion drops by placing the container at 25 °C. The initial cooling and heating rates in the emulsions were measured to be ≈0.4 K min^−1^ in the first 20 min and then gradually decreased to zero when the sample temperature approached that of the environment. After each step of cooling and heating, an emulsion specimen was placed in a glass capillary and the drop size distribution was determined microscopically or by DLS.

### Determination of the drop size distribution in emulsions

In most experiments, the drop size distribution in the emulsions was determined from microscope images, taken after each F/T cycle with × 100 microscope objectives. The accuracy in the measurements of the drop diameter by the microscopy method used is ±0.3 μm (ref. 69). For the experiments performed in capillaries, the drop diameters were measured one by one, using custom-made image analysis software. The diameters of more than 1,000 droplets in each sample were measured. For the bulk emulsions, the diameters of the droplets were measured by using Image Analysis Module of AxioVision Software. After each F/T cycle three independent samples were taken and for each of them more than 10,000 droplets were measured. The mean volume–surface diameter was determined from the relation 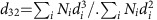, where *N*_*i*_ is the number of drops with diameter *d*_*i*_.

The drop size results, shown in Fig. 6c,d, were measured by DLS. These measurements were performed at 28 °C with 4700C system (Malvern Instruments, UK), equipped with a solid-state laser, operating at 514 nm. Multimodal software was used for the analysis of the autocorrelation function of the scattered light. The results shown in Fig. 6c,d are average from at least four measurements at scattering angles of 90° and 60° (very similar results were obtained at both angles). From the size-distribution histograms, by volume and by number, we determined the values of *d*_V50_ and *d*_N50_ (the medians in these distributions) and of *d*_V84_ and *d*_N84_ (the drop diameters, below which 84% of the emulsified material is present in volume or in number of drops for the respective cumulative distribution function^25^). The ratios *σ*_V_=*d*_V84_/*d*_V50_ and *σ*_N_*=d*_N84_/*d*_N50_ are convenient measures of the polydispersity for a log-normal distribution^25^. The values of ln(*σ*_V_) and ln(*σ*_N_) represent the half-width of the respective log-normal distribution curves^25^.

### Data availability

The data that support the findings of this study are available from the corresponding author on request.

## Additional information

**How to cite this article:** Tcholakova, S. *et al*. Efficient self-emulsification via cooling-heating cycles. *Nat. Commun.* **8,** 15012 doi: 10.1038/ncomms15012 (2017).

**Publisher's note**: Springer Nature remains neutral with regard to jurisdictional claims in published maps and institutional affiliations.

## Supplementary Material

Supplementary Information Supplementary Figures, Supplementary Methods and Supplementary Reference

Supplementary Movie 1 Drop shape evolution of hexadecane-in-water drops, stabilized by the nonionic surfactant C_18_EO_20_, at a cooling rate of 1.44 K min^-1^ and initial drop size d_ini_ ≈ 33 μm. One sees consecutive cycles of drop self-shaping and breakage events. The final observed shapes are platelets and ellipsoidal droplets, extruding thin fibers with diameter < 1 μm. Scale bar, 50 μm.

Supplementary Movie 2 Melting of a frozen trigonal platelet with long coiled protrusions. The system is a hexadecane drop (C16) in 0.38 wt. % C_18_H_37_SO_4_Na surfactant solution. Upon fibre melting, thousands of small droplets with diameter ≈ 1 μm are formed, due to a Rayleigh-Plateau instability, whereas the central triangular region melts into several big droplets. Scale bar, 20 μm.

Supplementary Movie 3 Melting of a frozen hexagonal platelet. The system is a pentadecane drop (C_15_) in 1.5 wt. % of C_16_SorbEO_20_ surfactant solution. Upon melting, hundreds of droplets are spontaneously formed, due to the process of crystal-melt fragmentation. Scale bar, 20 μm.

Supplementary Movie 4 Melting of frozen hexagonal platelets of hexadecane (C_16_) in 1.5 wt. % of C_18_SorbEO_20_ surfactant solution. Upon melting, each hexagonal platelet melts back into one to several liquid drops, viz. no intensive crystal-melt fragmentation is observed (compare with Supplementary Movie 3). Scale bar, 20 μm.

Supplementary Movie 5 Separation of natural fats into unsaturated (liquid) and saturated (solid) components. The surfactant mixture contains 0.05 wt. % (in total) of 7:3 by weight LAS:EO7 surfactants. The fat globules are composed of a 7:3 soybean oil:tristearin mixture. Scale bar, 20 μm.
