# Supplementary material for: Efficient self-emulsification via cooling-heating cycles
Source: Nat Commun. 2017 Apr 27;8:15012. doi: 10.1038/ncomms15012 (PMC5457670; doi:10.1038/ncomms15012)
Supplement: Supplementary Information — Supplementary Figures, Supplementary Methods and Supplementary Reference [file ncomms15012-s1.pdf]

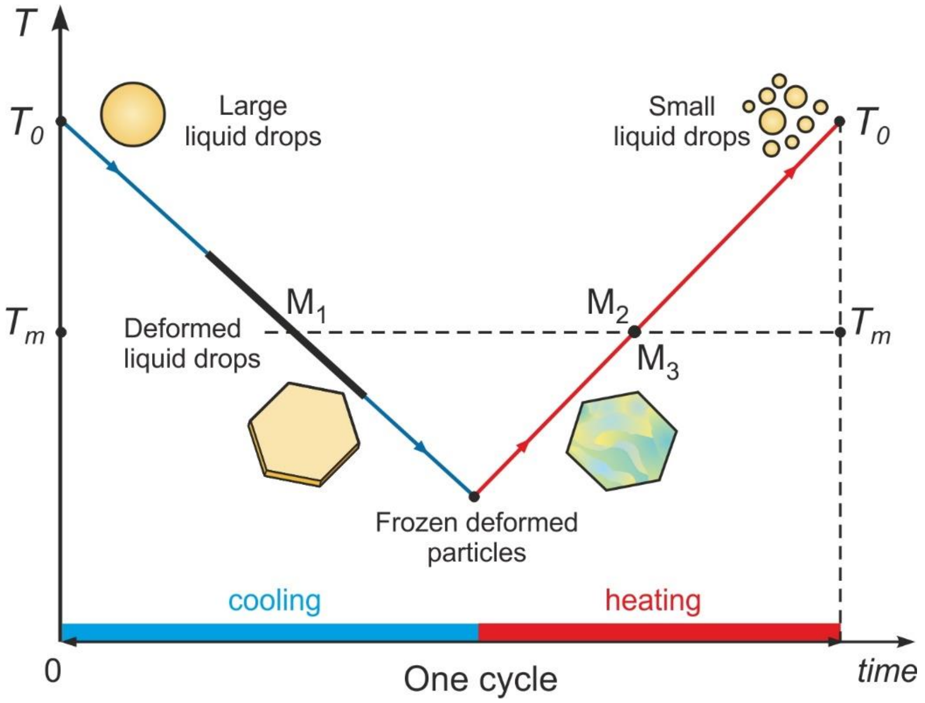

<u>Supplementary Figure 1.</u> Procedure for implementing the discovered self-emulsification method. Temperature cycling between freezing the droplets of a coarse emulsion and subsequent melting at the melting temperature of the bulk oil, T_m, leads to spontaneous droplet breakup via three distinct mechanisms ($M_{1\text{-}3}$). In M_1 fluid drops spontaneously burst during cooling, while in M_2 and M_3 frozen particles burst upon melting.

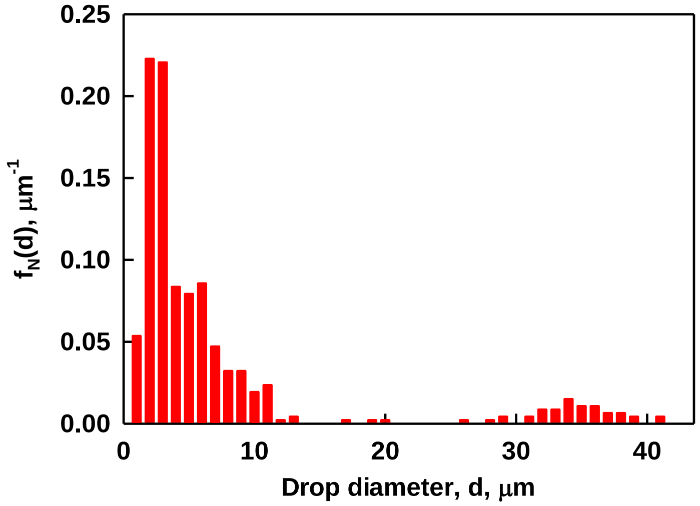

Supplementary Figure 2. **Drop-size histogram by number** for tetradecane (C_{14}) emulsion, stabilized by 1.5 wt. % $C_{18}EO_{20}$ surfactant, after one cycle of drop freezing and melting (one F/T cycle). One sees the formation of a bi-modal emulsion with two characteristic drop diameters – 35 μm (inherited from the initial emulsion) and 2.5 μm (as a result of fibre disproportionation upon fibre melting).

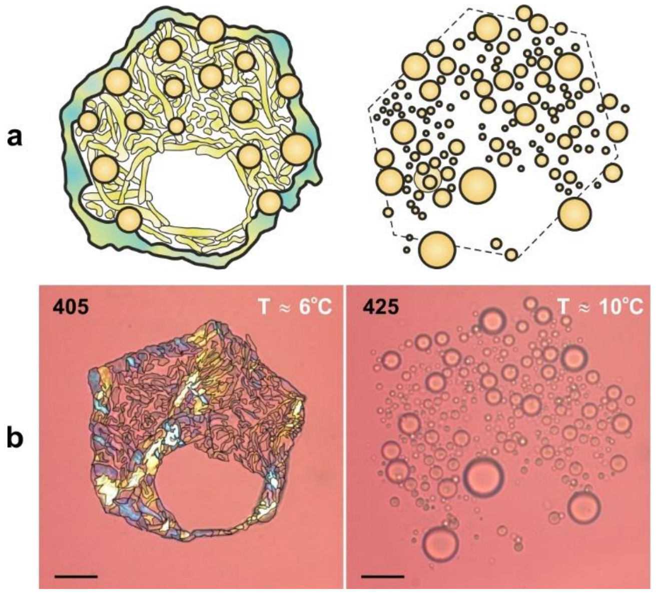

Supplementary Figure 3. **Process of fragmentation via melt-crystal dewetting. (a)** Schematic presentation; **(b)** Microscopic images illustrating this process for C_{15} platelets, stabilized by $C_{16}SorbEO_{20}$, **Supplementary Movie 3**.The numbers in (**b**) denote the crystal domains and the fragmented drops on the left and on the right image, respectively. Scale bars, 20 μm.

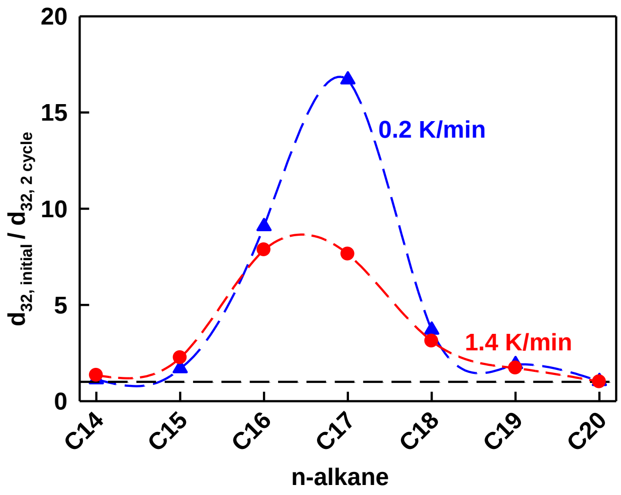

Supplementary Figure 4. Ratio of the initial mean drop diameter over the mean drop diameter after the second F/T cycle for oil-in-water emulsions prepared with different *n*-alkanes and stabilized by 1.5 wt. % $C_{18}EO_{20}$. Experiments are performed at a heating rate of 1.6 K min^{-1}, and at low and high cooling rates, 0.2 K min^{-1} and 1.4 K min^{-1}, respectively.

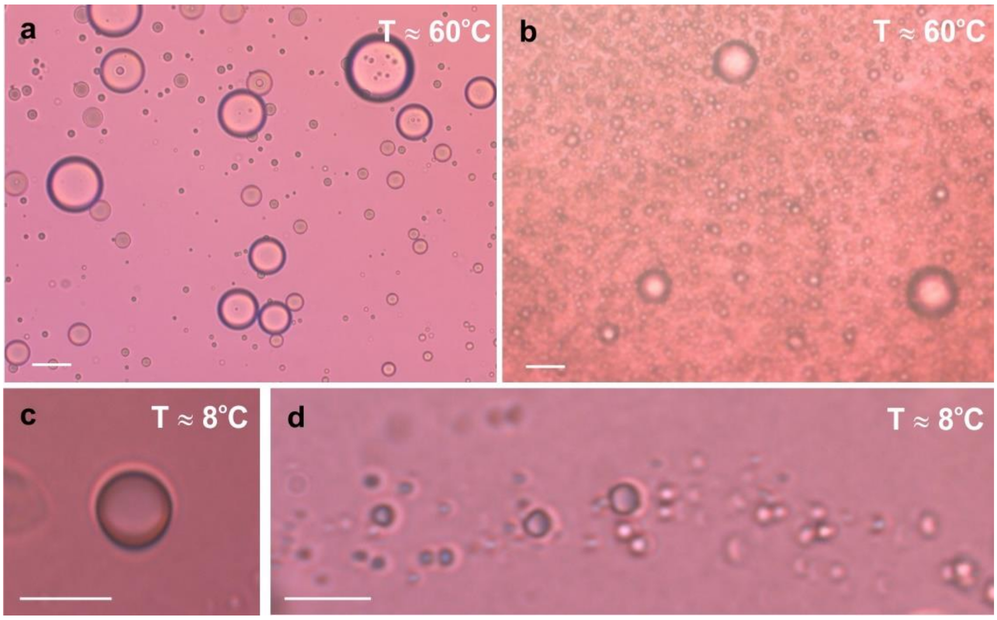

Supplementary Figure 5. Microscope images of self-emulsified drops. (**a,b**) trimyristin and (**c,d**) decanol before (a,c) and after (b,d) one cooling-heating cycle in a capillary (**Supplementary Fig. 2**). The drops spontaneously burst during the cycle by Mechanisms 1-3, described for alkanes. These emulsions are prepared with 1.5 wt. % $C_{18}EO_{20}$; the experiments are performed at a cooling rate of 0.5 K min^{-1} and heating rate of 1.6 K min^{-1}. Scale bars: 20 μm (a,b) and 10 μm (c,d).

Supplementary Methods

Matherials. Tetradecane (referred to as C_{14}), pentadecane (C_{15}), hexadecane (C_{16}), heptadecane (C_{17}), octadecane (C_{18}), nonadecane (C_{19}), eicosane (C_{20}), trimyristin (TM), tristearin (TS) and n-decanol were all products of Sigma-Aldrich with purity > 99%. Soybean oil (SBO) was obtained from a local store. The alkanes were used as dispersed oily phase in the emulsification experiments, whereas the tristearin and soybean oil were used for studying the mechanism of melt-crystal fragmentation (contact angle measurements and microscopic observations). The melting temperatures of the paraffins are: T_m = 5.4 °C for C_{14}, T_m = 9.9 °C for C_{15}, T_m = 18.2 °C for C_{16}, T_m = 22.0 °C for C_{17}, T_m = 28.2 °C for C_{18}, T_m = 32.1 °C for C_{19} and T_m = 36.8 °C for C_{20}[1].The hexadecane and soybean oil were purified from surface-active contaminations by passing them through a glass column, filled with Florisil adsorbent. The triglycerides, the other alkanes and the decanol were used as received.

Two series of nonionic surfactants with the commercial names Brij and Tween (all products of Sigma-Aldrich) were used as dispersion stabilizers. The Brij surfactants are polyoxyethylene alkyl ethers, which differ in the number of oxyethylene groups and the length of the alkyl chain. The following Brij surfactants were used: Brij 58 (polyoxyethylene-20 hexadecyl ether, $C_{16}EO_{20}$) and Brij 78 (polyoxyethylene-20-stearyl ether, $C_{18}EO_{20}$). Tween surfactants are polyoxyethylene-sorbitan alkylates, which have a sorbitan ring bound a hydrocarbon tail and to hydrophilic chains of 20 oxyethylene groups. The surfactants in this group differ in the length of the alkyl chain only. The series studied includes: Tween 40 (polyoxyethylene-20-sorbitan-monopalmitate, referred in the text as $C_{16}SorbEO_{20}$) and Tween 60 (polyoxyethylene-20-sorbitan-monostearate, $C_{18}SorbEO_{20}$). The nonionic surfactants were chosen to be water-soluble with high hydrophilic-lipophilic balance, HLB > 12, as such surfactants stabilize well the oil-in-water emulsions.

We used also two ionic surfactants – the cationic cetyltrimethylammonium bromide (CTAB, product of Sigma-Aldrich with purity ≥ 99%; $C_{16}TAB$) and sodium octadecyl sulfate (product of Merck with purity ≥ 99%; $C_{18}SO_4Na$).

To study the mechanism of crystal-melt fragmentation we used the surfactants sodium linear alkylbenzene sulfonate (LAS) and $alkyl_{13\text{-}15}$ polyoxyethylene-7 ether (EO7), both products of Stepan Co (Northfield, IL, USA).

All surfactants were used as received. For emulsion preparation, the emulsifier concentration in the aqueous solutions (1.5 wt. % for all nonionic surfactants, 0.37 wt. % for the anionic surfactant and 0.5 wt. % for the cationic surfactant) was chosen to be sufficiently high to suppress drop-drop coalescence during the experiments. All aqueous solutions were prepared with deionized water, which was purified by MilliQ or Elix 3 module (Millipore, USA).

Preparation of the initial emulsions. The initial hydrocarbon-in-water emulsions were prepared by a laboratory Microkit membrane emulsification module (Shirasu Porous Glass Technology, Japan) which works with tubular glass membranes of outer diameter 10 mm and working area of

approximately 3 cm[2]. Membranes with mean pore diameter of 2 μm, 3 μm, 5 μm and 10 μm were used for preparing initial emulsions with drop diameters around 6 μm, 10 μm, 17 μm and 35 μm.

Procedure for observation of TS-SBO layers placed in contact with surfactant solutions.
A sketch of the experimental setup is shown in **Supplementary Fig. 6**. Mixture of SBO:TS (7:3) was pre-heated and mixed at T = 70°C, and then deposited on dry pre-cleaned microscope slide. Upon cooling, the TS freezes in a layer, composed of a network of fat crystals, impregnated with liquid soybean oil (SBO). Afterwards, the slide was positioned over a Teflon ring in a vessel, which contains surfactant solution. The vessel was then placed in a thermostated cell and the experiments were performed at T = 25°C.

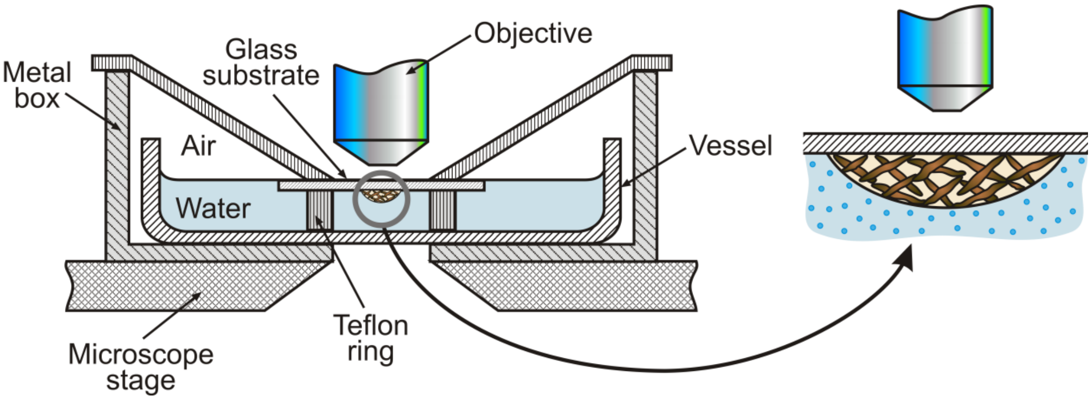

__Supplementary Figure 6.__ **Experimental setup for observation of TS-SBO layers.** Schematic diagram of the experimental cell for optical observation of the evolution of a mixed tristearin+soybean oil layer, placed in contact with surfactant solution.

Measurement of the contact angle of liquid oil over a substrate of solid fat. The contact angle of a liquid oil drop (SBO) placed on a flat substrate of solid fat (tristearin, TS) in the presence of different surfactant solutions was measured on a DSA 10 instrument (Krüss, Germany). The solid fat substrates were prepared by the following procedure: TS was melted at T = 80 °C and 100 μL were pippeted on a pre-heated hydrophobized glass slide. Then, a second pre-heated hydrophobized glass slide was used to sandwich the TS oil layer between the slides. The thickness of the TS layer (0.17 mm) was controlled by using microscope glass cover slips as spacers between the two glass slides. The layer was kept for 15 min at room temperature to solidify and then stored overnight at 5 °C in a refrigerator. Before the actual contact angle measurement, the cover glass slide was removed, yielding a clean and relatively smooth TS surface. Finally, the solid fat substrate was placed at the bottom of an optical cuvette, and a drop of SBO was placed on the TS surface. The contact angle of the liquid drop on the solid layer was determined by optical observation from the side (through the walls of the cuvette), before and after pouring surfactant solution in the cuvette.

Supplementary Reference

1. Small, D. M. *The Physical Chemistry of Lipids. From Alkanes to Phospholipids* (Plenum, New York; 1986).
